# The Tandem Reconnection and Cusp Electrodynamics Reconnaissance Satellites (TRACERS) Science Operations Center

**DOI:** 10.1007/s11214-025-01199-x

**Published:** 2025-08-11

**Authors:** I. W. Christopher, C. A. Kletzing, D. Crawford, C. Piker, D. Wilkinson, K. Steele, S. M. Petrinec, S. Bounds, S. Vaclavik, S. Omar, E. Shults, M. Winter, D. M. Miles

**Affiliations:** 1 Department of Physics and Astronomy, University of Iowa, Iowa City, 52245 IA USA; 2 Lockheed Martin Advanced Technology Center, Palo Alto, CA USA; 3 Millennium Space Systems, A Boeing Company, El Segundo, CA USA; 4 Nextage, LLC, Glenwood, MD USA

**Keywords:** TRACERS, Magnetic reconnection, Cusp, SOC

## Abstract

The primary purpose of the Tandem Reconnection And Cusp Electrodynamics Reconnaissance Satellites (TRACERS) Science Operations Center (SOC) is to ensure that the data necessary to achieve the TRACERS science goals are acquired, processed, and distributed to the scientific community. The SOC role in data acquisition is to facilitate science instrument planning and operations, through a weekly commanding cycle. Data processing includes generation of Level 0 and Level 1 data products, creation of Spacecraft Planet Instrument Camera-matrix Events (SPICE) kernels to provide spacecraft ephemerides and coordinate transforms for the mission, and ensuring consistency of all Level 2+ products produced by the individual instrument teams. Data distribution is undertaken in two ways. First, by hosting TRACERS data products on a public web portal during the active mission, and second by preparing mission data for transfer to the Space Physics Data Facility (SPDF) for long-term archiving.

## Introduction

The TRACERS mission, described in full in Miles et al. (2025a), is comprised of two identical spacecraft orbiting the Earth in a string-of-pearls, low-altitude, sun-synchronous, polar orbit that passes through or near the northern magnetospheric cusp region once per orbit. The primary goal of the mission is to use cusp-crossing observations made by the TRACERS Instrument Suite (TIS) on each spacecraft to determine whether variability of magnetic reconnection at the magnetopause is primarily temporal or spatial, with secondary goals of then revealing details of that variability. The TIS is comprised of five science instruments plus one Technology Demonstration instrument: ‘top-hat’ particle detectors for ions (Analyzer for Cusp Ions (ACI), Fuselier et al. 2025) and electrons (Analyzer for Cusp Electrons (ACE), Halekas et al. 2025); spin-plane electric field antennas (Electric Field Instrument (EFI), Bonnell et al. 2025); a fluxgate magnetometer (Magnetometer (MAG), Strangeway et al. 2025); a search coil magnetometer (Magnetic Search Coil (MSC), Hospodarsky et al. 2025) and a new design of fluxgate magnetometer (MAGnetometers for Innovation and Capability (MAGIC), Miles et al. 2025b). These mission goals rely on the acquisition and processing of high-quality, high-rate data from these instruments during up-to 15 cusp crossings per day, and the distribution of those data to the science community in a timely manner. The TRACERS Science Operations Center (SOC) is responsible for organizing and executing that process.

The SOC works closely with the TRACERS Mission Operations Center (MOC) at Millennium Space Systems, the company that built and operates the two spacecraft. Together, the SOC and MOC manage the flow of commanding to the spacecraft and of the resulting data from the spacecraft to the ground and then to the science community. The SOC consists of individuals and systems responsible for:

- Planning science operations.
- Preparing TIS commanding.
- Obtaining raw data from the MOC and processing it into Level 1 data products and Spacecraft Planet Instrument Camera-matrix Events (SPICE) kernels.
- Making Level 1 data products, and all necessary support data, available to the science instrument teams for further processing.
- Providing a Quicklook data interface for monitoring the state-of-health of the instruments.
- Receiving higher-level science products back from the science instrument teams, and making those products available to the broader science community through a web-based portal.
- Archiving all TRACERS data at NASA’s Space Physics Data Facility (SPDF).

**Fig. 1:**
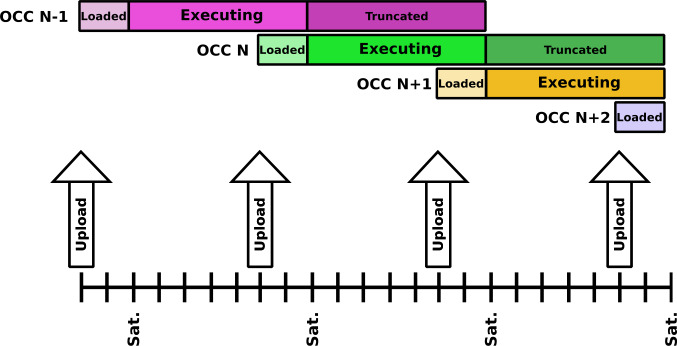
A schematic of the Operational Cadence Cycle, showing weekly uploads of 2-week blocks of commanding. During normal operations, the second half of each block is overwritten by the subsequent week’s upload

The first two items comprise the bulk of the data transfer from the SOC to the MOC, and form the heart of the Operational Cadence Cycle (OCC). This is the procedure which governs the operational flow from the SOC to the MOC, and subsequently to the spacecraft. The OCC is a weekly process, wherein the SOC prepares the planning and TIS commanding files covering a 2-week span of operations. These files are sent to the MOC by the middle of the week preceding the date upon which those activities should commence. From them, the MOC constructs the appropriate spacecraft commanding files, and then uplinks them on Thursday or Friday. Those commands then become active on Saturday, replacing any commanding that remained from the previous week’s OCC upload, as illustrated in Fig. 1. More details on this process are given when TIS Commanding is discussed, in Sect. 2.4. The goal is that after any OCC upload, the spacecraft will have all commanding necessary to act autonomously for up to two weeks, should there be any extended interruption of contact with the ground.

It should be noted that these activities are performed independently, but mostly in parallel, for each of the two spacecraft. It is only with the creation of the higher-level data products that data from the two spacecraft begin to be compared and combined, and thereby used to answer the mission’s science questions. Operationally, the main impact of the 2-spacecraft nature of the mission is in the choice of the length of time by which the trailing spacecraft lags the leading one along the orbit track. This lag will be varied between 10 and 120 seconds during the primary mission, but this variation will be planned by the science team and implemented by the MOC, with the SOC playing no direct role. As such, almost all of the discussion below will be couched in terms of a single spacecraft, with the implicit understanding that discussion could apply to either TRACERS-1 (TS1) or TRACERS-2 (TS2).

The remainder of this paper describes each of these activities. Section 2 covers planning and commanding topics, with Sect. 3 discussing the processing of the resulting data. Section 4 describes the SOC’s role in distributing those processed data products, and Sect. 5 summarizes how those data will be archived. A summary is provided in Sect. 6. Finally, an Appendix is provided that defines the reference frames used by the TRACERS mission.

## Operations Planning

### Targeting the Cusp

The science target of TRACERS is the northern magnetospheric cusp, which is normally near, or at least centered near, noon magnetic local time (MLT), and is at magnetic latitudes (MLat) between ~+60° and ~+85°. At any given time, though, the shape and location of the cusp can vary significantly, depending on the conditions in the upstream solar wind (Petrinec et al. 2025, and references therein). To maximize the number of actual northern cusp crossings during the nominal 1-year prime mission, a detailed analysis (Petrinec et al. 2025) was performed. A parameterized statistical model of the cusp location, using historical data, was constructed, and various test orbits were compared against it. The analysis showed that a circular, sun-synchronous polar orbit with a local time of descending node (LTDN) around 10:30 best achieves the desired optimization. The altitude of the orbit only weakly affects this analysis, at least within the low Earth orbit (LEO) domain of the mission design, so launch considerations and “orbit crowding” were the main drivers behind the selection of ~590 km as the orbit altitude (above a Mean Sea Level of 6371.2 km). Since the LTDN is a key part of the orbit design for the mission, the official orbit numbers for TRACERS will use the UTC time of an orbit’s descending node as the designated start for that orbit, with the first partial orbit after launch being designated as orbit number 0.

An operational constraint affecting cusp targeting is that the downlink telemetry budget only allows the acquisition of a few minutes (nominally 6 2/3) of full-rate TIS data per orbit. This requires that the TIS be commanded into, and then out of, full-rate mode once per orbit, at times that optimize the chance (on average) of encountering the cusp. Counterweighting this is the mission design requirement that TRACERS operations be simple and low-overhead, which precludes doing detailed analyses and complex commanding for each orbit. Instead, the orbit analysis indicated that it would be sufficient to simply define the science region of interest as the crescent defined by 06:00 to 18:00 MLT and +60° to +85° MLat, and to toggle the TIS into full-rate mode for a given spacecraft whenever it enters that crescent, and off when it exits. The portion of the orbit when the TIS is in this mode is called the Region of Interest (ROI), while the remainder of the orbit, when only decimated TIS data are stored for later downlink, is called the Backorbit (BOR).

### ROI Determination

**Fig. 2:**
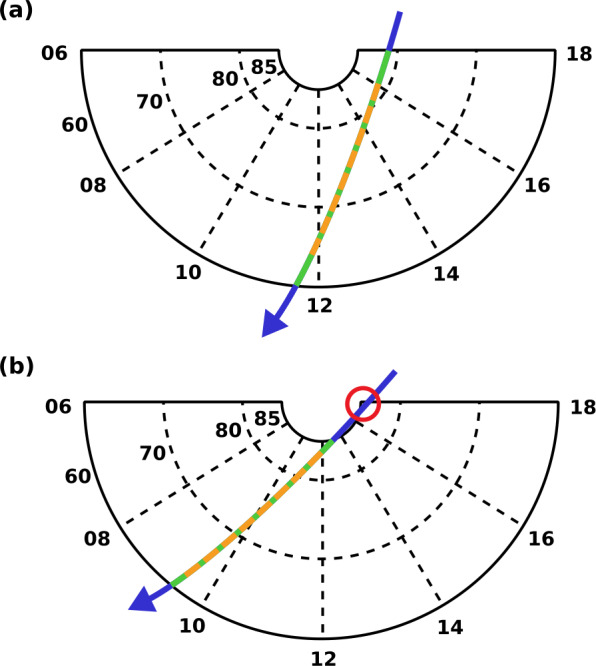
(a) An example spacecraft track (blue) overlaying a geomagnetic grid of MLat and MLT, viewed from above the north geomagnetic pole. The solid curve enclosing the grid denotes the science region of interest. The part of the track shown in green is within that region, while the part shown in dashed orange is the optimized ROI selected by the planning software. (b) Same as (a), except for an orbit where the spacecraft track enters and exits the science region of interest twice. The red circle indicates the first such encounter, which is discarded by the planning tool

The process of determining the actual ROI start/stop times used for each orbit begins with the generation of predictive orbit ephemerides at the MOC. These orbit predicts are calculated from the definitive as-flown ephemerides calculated onboard the spacecraft by the Guidance, Navigation and Control (GNC) subsystem (Omar and Reynolds 2024), using Global Positioning System (GPS) data, and downlinked on a daily basis. The predicts are projected forward, at a 10-second resolution, for four weeks, which is more than sufficient for normal operations planning, while also being at the edge of what is meaningful for an orbit at the TRACERS altitude, given the unpredictable effects of atmospheric drag. The SOC then uses these predicts to calculate the magnetic coordinates (MLat and MLT) for the spacecraft throughout each orbit. For each day, these coordinates are filtered against the 06:00 to 18:00 MLT and +60° to +85° MLat science region of interest, obtaining a first cut of the ROI times for each orbit of that day. The result is the green segment of the representative orbit track shown in Fig. 2a. The ROI algorithm calculates and assesses the ROI times on a per-day basis, as this enables calculation of a daily-average ROI length. If this average exceeds, or is noticably under, the allotted 400 seconds per orbit of full-rate data accumulation, then the MLat/MLT bounds are contracted or expanded, respectively, and the day’s ROI times are recalculated. The algorithm repeats this process until final ROI times are obtained that result in a daily average length that is near, but not over, 400 seconds. The result of this optimization is shown by the orange dashed segment of the track in Fig. 2a.

Due to the “Pac-Man” shape of the science region of interest, and the fact that the spacecraft track across this region varies as the geomagnetic pole moves relative to the orbit plane as the Earth rotates daily, there are rare orbits where the spacecraft can enter and exit this region twice in a single orbit. For such orbits, the ROI determination algorithm selects the start/stop pair with the best chance of encountering the cusp. Figure 2b shows an example of this, with the unselected part circled in red. These ROI start/stop times, one pair per orbit, are calculated for a 2-week span, as part of the weekly OCC package that is provided to the MOC.

In addition to this a priori ground-based determination of the ROI times for each orbit, the GNC subsystem onboard the spacecraft also has a real-time capability to determine the entry and exit times for the science region of interest. This capability was created as a contingency against ground-calculated ROI times becoming “stale”. There was concern that if ground contact was lost for an extended duration, sufficient perturbations to TRACERS low-altitude orbit could accumulate over the operations planning window to materially affect the accuracy of the start/stop times. The algorithm used is similar to the ground-based one, in that the current spacecraft position is converted to MLat and MLT and then compared to the science region of interest. Since the process is real-time, however, there can be no optimization stage. As a result, this process triggers the start of ROI mode as soon as the spacecraft enters the 06:00 to 18:00 MLT and +60° to +85° MLat region, and ends the mode when it leaves the region, or after 400 seconds, whichever comes first. Since this operating mode can result in a less optimized targeting of the cusp, it is envisioned that it will be used as a backup, which could be set to enable automatically if any extended period of interruption in commanding uploads results in a risk of “stale” ROI data being used.

### Spacecraft Attitude Pointing

Aside from determining the ROI time interval for each orbit, the other main planning task for the SOC is determining the appropriate attitude for the spacecraft. Simplification of elements of the TIS design (Fuselier et al. 2025; Halekas et al. 2025; Bonnell et al. 2025) was enabled by requiring that the spacecraft spin axis be no more than ~25° from the direction of the Earth’s geomagnetic field during the cusp crossings. For TRACERS, the 14th edition of the International Geophysical Reference Field (IGRF-14) field model (Alken et al. 2021) is used to represent the Earth’s geomagnetic field for all operational planning purposes. At the TRACERS altitude, there is no need to add external field models to obtain sufficiently accurate field vectors (Petrinec et al. 2025). As such, the SOC uses IGRF-14 B-vectors calculated along the ROI portion of the predicted orbit track to derive a pointing direction for the spacecraft.

In the ideal case, the spacecraft would be reoriented once per orbit to align its spin axis with the IGRF-14 direction at the point within that orbit’s ROI that is closest to the projected cusp. With ~15 orbits per day however, that would add complexity to spacecraft planning and operations—complexity that is beyond the scope of TRACERS. Instead, the analysis of Petrinec et al. (2025) determined that it would not be necessary to realign the spacecraft to the model field for every orbit, but instead would be sufficient to do so only once per day. At the core of this process is the concept of a Local Aggregate B-Field (LABF), which is effectively an average of all the IGRF vectors that a spacecraft would encounter during all of the ROI passes for a given day. With that spacecraft reoriented to that LABF vector during the first orbit of that day, the spin axis will be optimized to best align with the cusp magnetic field for all of that day’s ROIs, and for the majority of them is expected to be within 10-15° of that field direction.

This LABF is calculated by first using the algorithm described in Petrinec et al. (2025) to average the IGRF-14 vectors along each of the 15 ROIs of the day to produce an LABF_ROI vector for each. These 15 vectors are themselves then averaged using the same algorithm, to produce the LABF vector for the day as a whole (referred to as LABF or LABF_Day). The resulting list of alignment vectors, one for each day in a 2-week span, is then provided to the MOC as part of the OCC package. The MOC then uses these to construct the appropriate commanding for the spacecraft GNC system.

### TIS Commanding

By mission design, minimal TIS commanding is required during normal operations. The instruments all essentially have just a single operating mode—once they are activated, they flow data to the TIS Central Data Processing Unit (CDPU) at their full data rate. In ROI mode, the CDPU compresses these data, packages the result into packets and flows them to the spacecraft data handling system, where they are stored until being downlinked. In BOR mode, the only difference is that the CDPU decimates the data it receives from the instruments before compressing them. Therefore, some instruments will need no commanding most weeks, while others will require only calibration sweeps, or similar activities. Section 2.2 above describes how the times of the transitions into and out of ROI mode are determined. The actual trigger for that transition is not a TIS command, but a spacecraft bus command that is sent to the CDPU. Those command sequences are constructed by the MOC, using the ROI times provided by the SOC, and are not part of the TIS commanding package created by the SOC.

The simple design of the TIS also removes any need for a real-time commanding capabliity at the SOC, as any serious instrument anomalies can be dealt with by simply turning off that instrument. These can then be investigated and dealt with during regular ground contacts using the MOC’s real-time commanding capability. That capability is also used for any TIS engineering activities that require a real-time connection. Nominally there is expected to be one or two ground contacts per day, allowing multiple opportunities per week for any such special commanding.

Routine TIS commanding will consist mainly of calibration, maintenance and engineering activities. TIS personnel provide any such activity requests to the SOC, where they are turned into command files as part of the weekly OCC process. Since many of these activities involve commands that are repeated regularly, extensive use is made of stored and pre-validated command sequences, further simplifying the commanding process. At the MOC, the TIS commanding is integrated and deconflicted with the spacecraft bus commanding for that OCC, resulting in a Deconflicted Operations Plan (DOP). The DOP is then sent back to the SOC for review, and upon concurrence from the SOC that the combined commanding is satisfactory, the DOP package is then prepared for uploading to the spacecraft.

The transition from one OCC to the next is planned as part of this deconflicting process. The DOP is inspected by the MOC to determine the appropriate time on Saturday for the transition from the current OCC to the new one to occur. The time is selected such that no TIS or bus command activity is executing, nor is the spacecraft within an ROI. A bus script is then constructed that will delete all of the current OCC’s files on the spacecraft that are scheduled to execute after the designated time. After the full commanding package has been uploaded and verified, this script will execute to remove the designated commanding from the current OCC, and to then unpack the new commanding in its place.

## Data Processing

The general flow of TIS science and housekeeping data is that each science instrument sends its data to the CDPU within the TIS, which packages and forwards the data to the spacecraft bus for storage. The stored data are then downlinked daily (nominally) to NASA’s Near Space Network (NSN). From the NSN, the data are transferred to the MOC, where the original data created by the CDPU are reconstructed from the various transfer formats. Files of these data are transferred to the SOC, where the data are parsed and stored in a PostgreSQL database as Level 0 (L0) data. The data are then processed by the SOC into two forms of Level 1 data (L1a and L1b), which are written to Common Data Format (CDF) files. These CDF files are the data-of-record for TRACERS, and form part of the mission archive. All CDF files for the mission will be fully self-documenting with ISTP-compliant metadata. The science data at this level are also available on-demand to the instrument teams via a secure Quicklook web interface (Sect. 3.6). The individual instrument teams then use the L1 data CDFs to create higher-level data products, which are subsequently sent back to the SOC for basic validation and then distribution to the science community and the mission archive. The L0 and L1a/L1b data are for internal use and are not distributed beyond the TRACERS team, while the L2 and higher data are the scientifically useful data products and are distributed to the scientific community in compliance with NASA’s open data policies.

Spacecraft bus housekeeping data follow a similar path from the bus to the MOC. Most of these bus data are then provided to the SOC for inclusion in the TRACERS mission archive. Select data that are pertinent to TIS or science operations are also processed into other formats, including the SOC database, where they can be access via the same Quicklook interface used for the L1 science data.

### CDPU Processing and TIS Telemetry Flow

Each TIS instrument constantly flows packet streams of science data to the CDPU throughout an orbit, regardless of whether the spacecraft is in ROI or BOR. The processing of those streams varies by instrument, and also by whether the CDPU is in ROI or BOR mode. The core data processing task of the CDPU is common to all instrument’s data however. This task is to compress the science data, pack it into Consultative Committee for Space Data Systems (CCSDS) telemetry packets, encapsulate these packets into Instrument Transfer Frames (ITF) (which provide synchronization and cyclic redundancy check (CRC) error-checking information) and then transmit these ITFs to the spacecraft data system for storage in solid state memory. We will refer below to these common steps as the compress-pack-ship processing. As part of the creation of each CCSDS packet a timestamp is applied to the packet, but discussion of this step is deferred until Sect. 3.2.

Turning to specific instruments, we begin with ACE and ACI processing, as theirs is the most straightforward. When the CDPU is in ROI mode, it simply does the basic compress-pack-ship processing. It takes the incoming data packets, amalgamates the contents into complete energy-angle sweeps of the sensor, compresses those science data and packs the result into CCSDS packets. In BOR mode, the incoming streams are first downsampled by discarding all but one out of every 128 sweeps before compressing the data for the remaining sweep and packing it into a CCSDS packet. In either case the packets are then wrapped in ITFs and sent to the spacecraft. For the MAG and MAGIC data, the process is similar except that the input is a simple, steady stream of magnetic field vectors, with no need for any amalgamation, and for a change in the BOR downsampling. For these data, the CDPU first applies an 8-point boxcar filter to the full-rate data stream before downsampling that modified stream. For both MAG and MAGIC, the downsampling rate is 1 in 8.

For EFI and MSC (whose data are routed through the EFI Main Electronics Box (MEB)), much more of the processing is instead performed by the EFI’s own Signal Processing (EFI_ESP) board, before the data are sent to the CDPU. Also, since EFI produces several different data types, there are multiple data streams produced, each undergoing distinct processing. Common to all these streams is that their data are already packed into CCSDS packets prior to leaving the EFI MEB. These packets contain uncompressed data however, so when the CDPU processes these data, it will create new CCSDS packets to contain the compressed version of the data. The timing and sequence information from the original packets are retained though, and copied to the new packets. For all EFI-sourced packets, this repackaging is incorporated into the standard compress-pack-ship processing.

The EFI DC voltage (VDC) and electric field (EDC) data types each result in two packet streams, one to be used in ROI mode and the other in BOR mode. The former streams contain full-rate data, while the latter contain data that has been downsampled by discarding all but one out of every 16 data samples. When these four streams are received by the CDPU, it discards the two BOR streams if in ROI mode, or discards the ROI streams when in BOR mode. The surviving two streams then undergo the compress-pack-ship processing.

The EFI AC electric field (EAC) and the MSC AC magnetic field (BAC) data types each result in just a single CCSDS packet stream. The raw data stream for each data type consists of a continuous 2048 samples/s, which is divided into 1024 samples per packet. In ROI mode, when the CDPU receives these streams, it simply does the compress-pack-ship processing on every packet. In BOR mode, the CDPU discards all but one out of every 128 packets, and then compress-pack-ship processes the remaining one. The result is a 1024-sample snapshot from the original data stream every 64 seconds.

The EFI high frequency AC electric field (EHF) data type results in a single CCSDS packet stream. The raw data for EHF is produced at $20 \times 10^{6}$ samples/s, but only a single 16384-sample (~82 ms) snapshot every 16 s is processed into packets by the EFI_ESP board. Each such snapshot is divided into 16 CCSDS packets, which are streamed to the CDPU. In ROI mode, the CDPU simply does the compress-pack-ship processing on this full stream of packets. In BOR mode, the CDPU keeps and does compress-pack-ship processing on only 1 set of 16 packets out of every 128, resulting in one snapshot being produced every 2048 seconds.

These science packets from all of the instruments flow over a single high-speed interface between the TIS and the bus data handling system. Since packets from all instruments are normally being produced nearly continuously, there is a mix of all packet types intermingled in this single flow. TIS housekeeping data are similarly packetized, and flow both over the same interface as the science data, and also over a second, low-speed connection. In the bus processing system, each of these two flows is accumulated in to a series of files, which are stored, awaiting transmission to the ground during the next downlink. A small number of the housekeeping packets flowing over the low-speed interface are also decoded and processed by the bus, for real-time monitoring of the state-of-health of the TIS.

All of the data products discussed above are summarized in Table 1. During ground contacts, the files of these data that have been saved to the spacecraft data system are packaged into further layers of packets and transfer frames for downlink to the NSN. At the NSN, the transfer frames that are received are stored in files, for later transfer to the MOC. At the MOC, the data are successively unpacked from their accumulated transfer mechanisms until files of the original ITF frames are obtained. Any frames lost during the downlink process are identified and scheduled for retransmission during a subsequent contact, although only a limited number of attempts will be made to recover such data. Finally, these files are transferred to a secure server at the SOC via Secure File Transfer Protocol (SFTP), where they are processed into L0 data. For archival purposes, the files of ITF frames are saved in their original form as received from the MOC.

**Table 1:** Data rates and Backorbit downsample factors for the fundamental science products produced by each of the TIS instruments

Instrument	Data product	Data rate in ROI	Downsample factor in BOR
ACE	Electron counts as a function of energy and angle	20 sweeps/sec	1/128
ACI	Ion counts as a function of energy and angle	~3 sweeps/sec	1/128
EFI	DC Voltages	128 samples/sec	1/16
	2D DC Electric Field	128 samples/sec	1/16
	2D AC Electric Field	2048 samples/sec	1/128 (1 × 1024–sample snapshot per 64 s)
	1D High Frequency Electric Field	20 × 10^6^ samples/sec (1 × 16384–sample snapshot per 16 s)	1/128 (1 × 16384–sample snapshot per 2048 s)
MAG	3D DC Magnetic Field	128 samples/sec	1/8
MSC	3D AC Magnetic Field	2048 samples/sec	1/128 (1 × 1024–sample snapshot per 64 s)
MAGIC	3D DC Magnetic Field	128 samples/sec	1/8

### L0 Processing

L0 processing begins with the files of ITF frames received from the MOC. The CRC provided by each frame is used to check for corruption of the CCSDS packet within that frame. Packets that fail this check are logged, but no further processing occurs for them. During normal processing, no attempt is made to recover such data. For each valid packet, the instrument and data type are identified using a TIS ID code and a CCSDS Application ID (APID) encoded within the CCSDS packet. Any duplicate packets that are received are discarded. Each packet has been tagged with a POSIX timestamp, recording the time when that packet was processed by the CDPU. That POSIX time encodes a GPS time produced by the spacecraft central processor and is supplied to the TIS via a Pulse-Per-Second (PPS) signal. From this POSIX timestamp the L0 system constructs a CDF TT2000 timestamp. In cases where it is known that the times provided by the spacecraft are not synced with true GPS (e.g., when the receiver on the spacecraft loses GPS lock), TIS and spacecraft housekeeping data will be used to adjust the affected CDF TT2000 timestamps to be as accurate as the available information allows. In particular, several instruments report an offset to the PPS timing signal within their own metadata, which can be used as an additional time reference. All such instances requiring time corrections will be logged. If further analysis is ever required for any timing issues, the raw POSIX timestamp is always available within the raw CCSDS packet in the database, and also within the L1a CDF files produced by the subsequent processing step. With each valid packet now having been timestamped and identified by data source, it is added to the PostgreSQL database that is the heart of the SOC L0/L1 data system. The primary access key for each packet within this database is the TT2000 timestamp for that packet, which has the useful side-effect of automatically reordering any packets that were out-of-sequence upon arrival at the SOC.

### L1 Processing

Almost all level 1 processing is handled by an early version of the open-source *das3* multi-mission software suite. The suite is built around a PostgreSQL database that ingests raw data streams, processes them in configurable ways, and produces science-ready output files. Mission-specific details of the processing (conversion of binary data, calibration rules, etc.) are controlled by configuration data structures that are stored separately from the science data within the database. The system also has a plugin interface to handle custom processing tasks. For instance, TRACERS uses plugins tied to each data type (APID) to handle data-specific decompression. This unified structure easily allows automatic reprocessing of L1a and/or L1b data products whenever there is a change in the processing rules—for example a changed calibration table or a revised decompression module.

Level 1 processing on TRACERS results in two distinct sublevels of data products, L1a and L1b. Each is the result of applying select steps from a multi-stage process:

1. Decompression of packet payload data.
2. Conversion from raw binary data to integer engineering values.
3. Creation of timestamps for individual data records.
4. Calibration into physical quantities with units.
5. Rotation into geophysical reference frames (for vector quantities).

Processing through steps 1-3 results in L1a data, while adding the processing in steps 4-5 produces L1b data. In either case, the resulting data are written to daily CDF files for each APID, and can also be streamed to the Quicklook interface, or to other data visualization tools.

L1a begins with decompression of the science data stream that was packed into the CCSDS packets by the CDPU. As each type of data (e.g., magnetic field waveforms or ion energy-angle sweeps) has its own particular structure, distinct compression/decompression algorithms, implemented as *das3* plugins, are used for each. Many of these are built upon lessons learned from the compression of similar Van Allen Probes data. Note that TIS housekeeping data (which have distinct APIDs) are generally not compressed onboard the spacecraft, so no decompression step is necessary for these. Once the structure of the original binary packet stream from an instrument is recovered, those bits and bytes are converted to integer values representing the raw sensor output. Finally, timestamps are calculated for each individual record (e.g. a single MAG vector or ACI sweep) by propagating the packet-level TT2000 timestamp using APID-specific rules. The result is a coherent set of TIS data records that are individually timestamped, but are still in uncalibrated engineering units.

L1b processing then takes this set of L1a data and applies first-order, static and reversible calibrations to them, to produce data that are ready for science or engineering uses. These calibrations, again APID-specific, take the form of polynomial coefficients or lookup tables that are stored within the *das3* database. It is planned that these calibrations will be set prior to or during the commissioning phase of the mission, and will remain unchanged thereafter, allowing easy recovery of the original uncalibrated values if needed for subsequent data analysis. It is important to note that these are not the final calibrations necessary to produce high-precision science data. Those calibrations, many of which will be regularly revised as instrument data are accumulated and analyzed through the mission, will be applied during Level 2+ processing.

Processing at the L1b level also identifies all values that represent vector quantities, and performs frame transformations as needed. Each TIS sensor has a dedicated instrument reference frame, all specified relative to the spacecraft’s body-fixed rotating frame, the TRACERS Satellite Coordinate System (TSCS). Vector data are transformed into this TSCS frame, and also into a de-spun frame, called TRACERS Spin Sun (TSS), that uses the spacecraft spin axis and the spacecraft-to-Sun vector as its defining vectors. A summary of the reference frames used on TRACERS is provided in the Appendix. All frame transformations on TRACERS are performed using the SPICE system, described in the following section. The *das3* system includes a generic module to perform such SPICE transforms.

The normal latency for production of L1a and L1b CDF files is <1 day from receipt of the raw telemetry at the SOC. L1b is the highest level of processing that TIS housekeeping data are normally subjected to.

### SPICE Kernels

All reference frame transforms on the TRACERS mission are done using the Navigation and Ancillary Information (NAIF) Spacecraft Planet Instrument Camera-matrix Events (SPICE) system. This system consists of a software library plus a variety of kernel files that contain ephemeris, orientation and time information for spacecraft and solar system bodies. TRACERS-specific values for each spacecraft are contained in SPK (Spacecraft and Planet) kernels containing the ephemeris data, CK (Camera-matrix) kernels containing the attitude data, and SCLK (Spacecraft CLocK) kernels containing the mapping between the spacecraft clocks (which use UTC derived from GPS time) and the Barycentric Dynamical Time (TDB) that SPICE uses internally. Each spacecraft also has a CK kernel that is constructed to provide an IGRF-14 field-aligned coordinate system for that spacecraft. In addition, there is a kernel defining common geophysical reference frames, and one for each spacecraft defining various spacecraft-specific frames (Appendix summarizes these frames). These kernels are summarized in Table 2.

**Table 2:** Summary of the SPICE kernels produced for TRACERS. The Data Source column lists the source of the key input data for each product

Task	Kernel type(s) used	Spacecraft specific?	Data source(s)
Ephemeris	SPK	Yes	GNC
Attitude	CK	Yes	GNC
Spacecraft Clock	SCLK	Yes	GPS
Field-Aligned Frames	CK, Frame	Yes	IGRF-14
Spacecraft Frames	Frame	Yes	Engineering documents, GNC
Geophysical Frames	Frame, CK	No	IGRF-14

The data for the spacecraft ephemeris and attitude kernels come from the spacecraft GNC system, which uses GPS and onboard sensors to compute accurate position and attitude solutions at a ~1-second resolution. These data are downlinked with the spacecraft housekeeping and provided to the SOC by the MOC as GNC history files. These data are processed into the required SPICE kernels by the SOC as soon as the data are available, so that the kernels are available for L1b processing. The full SPICE kernel set is also made available to the instrument teams, where it is used for Level 2 (L2) and higher processing.

### Spacecraft Housekeeping Data

Spacecraft housekeeping data are processed by the MOC. Many of these data are then provided to the SOC, in the form of JavaScript Object Notation (JSON) files, both for archiving and for use in TIS-related analyses. Data pertinent to the TIS include current, voltage and temperature monitors for the TIS Main Electronics Board, ACI and ACE, as well as GNC data such as times of thruster firings and of magnetorquer activity. These data are processed, using a *das3* plugin, and added to the PostgreSQL database, where they can be accessed alongside TIS science and housekeeping L1 data in the Quicklook interface.

### Quicklook Interface

Timely access to instrument science and housekeeping data is critical to monitoring the health of those instruments. To facilitate this for the instrument teams, the SOC provides both static plots and a web-based tool for Quicklook access. The web tool allows team members to interactively plot any combination of variables that are available from the SOC telemetry database. These data are flowed on-demand from the *das3* system using the *das* streaming format, which is an open format usable by multiple data visualization packages. One such package is Autoplot (Faden et al. 2010), which is the tool generally used when making the TRACERS static plots. These are preconfigured Portable Network Graphics (PNG) plots, each designed with input from the instrument teams, and covering the timespan of one orbit (approximately 90 minutes). They can be automatically generated by the *das3* system when new data are processed, and are placed on the TRACERS Portal for the instrument teams to access.

### L2+ Data Products

The SOC does not create any data products above L1, as these are all made by the science instrument teams. However, the SOC is responsible for receiving those products back from the teams and validating the metadata and structure of the CDF files, to ensure consistency across all data products. A summary of all the TRACERS data levels is shown in Table 3.

**Table 3:** Summary of the data level designations for TRACERS data products. The Description column briefly summarizes the processing done at each level. The Expected Latency is the nominal time for the data product to be produced, after the time-of-receipt of the raw data at the SOC. This is a condensed form of the similar table in Miles et al. (2025a)

Level	Description		Expected latency	Distribution
L0	•	Received CCSDS packets with duplicate/corrupt packets removed.	<1 Day	TRACERS Internal
L1a	•	Unpacked and ordered data from CCSDS packets.	<1 Day	TRACERS Internal
	•	All data in as-received engineering units in instrument frame.		
	•	Best known timestamps on each sample.		
	•	All relevant CCSDS header metadata included from L0.		
L1b	•	Pseudo-physical units based on static and reversible calibrations.	<1 Day	TRACERS Internal
	•	Known lost/corrupted data logged.		
	•	Spacecraft (TSCS) frame and de-spun (TSS) frame.		
	•	No convolution of data from multiple instruments.		
L2	•	Calibrated physical units (via in situ calibration if applicable).	<2 Weeks	Public
	•	Spacecraft and geophysical frames (including magnetic field aligned).		
L3	•	Derived by irreversibly combining data from multiple instruments and/or multiple spacecraft.	<1 Month	Public
	•	Derived by irreversibly transforming or reducing data using physics.		
L4	•	Derived by irreversibly integrating data from models.	TBD	Public

## Data Distribution

One of the most important roles of the SOC is to be the distribution hub that disseminates the TRACERS science data to the wider science community. Equally important is the distribution of internal TRACERS data to the TRACERS team. Both tasks are fulfilled through a single web portal, at: https://tracers-portal.physics.uiowa.edu

Access to the internal portion of the Portal requires user authentication and allows TRACERS members access to operations and planning tools, SPICE kernels, L0 and L1 data, and the Quicklook interface. The remainder of the Portal is open for public access. It provides access to L2 and higher data products, organized by spacecraft and date. These data files will follow this general naming template:

tsN_lL_instrument_data_YYYYMMDD_vX.Y.Z.cdf

where “N” is the spacecraft (1 or 2); “L” is the data level (2, 3 or 4); “instrument” is one of ACE, ACI, EFW, MAG, MSC or MAGIC; “data” is one or more tags describing the data product; “YYYYMMDD” is the date; and “X.Y.Z” is the data version. The convention used when incrementing versions is that a change in X indicates a change in file structure, so software that processes the CDF may need to be updated. A change in Y indicates that changes were made that affect the science content of the file, so any derived science products should be reprocessed. A change in Z indicates that the file has been regenerated due to changes that do not reach the threshold for an X or Y increment (adding new data to an existing day file, minor changes to metadata, etc.). As such, downstream science reprocessing of existing products may not be necessary, although it is generally a good idea to do so anyway. Some of the key L2 data products are shown in Table 4.

**Table 4:** Example names for select L2 data products

Instrument	Filename	Key data product
ACE	ts1_l2_ace_def_20250801 _v1.0.0.cdf	Electron differential energy flux
ACI	ts1_l2_aci_def_20250801_v1.0.0.cdf	Ion differential energy flux
EFI	ts1_l2_efi_edc_20250801_v1.0.0.cdf	Perpendicular DC electric field
	ts1_l2_efi_eac_20250801_v1.0.0.cdf	Perpendicular AC electric field
MAG	ts1_l2_mag_bdc_roi_20250801_v1.0.0.cdf	Vector DC magnetic field in ROI
	ts1_l2_mag_bdc_bor_20250801_v1.0.0.cdf	Vector DC magnetic field in BOR
MSC	ts1_l2_msc_bac_20250801_v1.0.0.cdf	Vector AC magnetic field

One key product available on the Portal will be a list of validated cusp crossings. The SOC and the instrument teams will use the L1 data to determine for every orbit if the cusp was encountered. The primary criteria is the appearance in the ACI data of non-negligable ion flux at around 400 eV. Such flux is almost entirely absent in the polar cap and ring current regions that border the cusp. The valid crossings will be tabulated, along with support data (e.g., solar wind data) for that event. A User’s Guide describing how the TRACERS Portal is organized and how to best utilize it is prominently available at the website.

## Archiving

The archive for the TRACERS mission will be at NASA’s Space Physics Data Facility (SPDF): https://spdf.gsfc.nasa.gov

It will contain all science data products generated during the lifetime of the mission. These products will be available for archiving at the same time that they are available on the Portal and are released (for the higher level products) to the public. The calibration data used to make these science products will also be included in the archive, along with a calibration history, thus allowing the provenance of any given data product to be determined. The operational history of the mission will be archived, including ephemerides, SPICE kernels, event list, anomaly logs, etc., as will all other data necessary to interpret the science results of TRACERS. The archiving process itself will be executed by the SPDF, as they will pull data from the TRACERS Portal as it becomes available.

## Summary

The TRACERS SOC is a lean, efficient organization that performs the operational planning and low-level data processing needed to ensure the success of TRACERS mission. A streamlined weekly planning process maximizes the likelihood of capturing the high-quality science data required to answer the mission’s science questions. Rapid processing of these raw data into the products necessary for that science analysis follows receipt of those data. Finally, those science results are gathered at the SOC and made available for broader consumption and for archiving.

## Appendix: TRACERS Reference Frames

TRACERS makes use of several standard geophysical reference frames, as well as a number of spacecraft-centered frames, all of which are defined using SPICE frame kernels. Below is a summary of each of these frames. Where they can aid in understanding the frame, brief implementation notes are also included.

### A.1 Geophysical Frames

#### A.1.1 Geocentric Equatorial Inertial (GEI) Frames

The Geocentric Equatorial Inertial frames are a set of reference frames that have their X axes pointing from the Earth towards the first point of Aries, which is the position of the Sun at the vernal equinox. The Z axes are parallel to the rotation axis of the Earth.

**Table:**

X_GEI_	Direction of from the Earth to the first point in Ares (the position of the Sun at the vernal equinox).
Z_GEI_	Along the spin axis of the Earth, pointing northward.
Y_GEI_	Z_GEI_ × X_GEI_.

The individual frame implementations vary because the position of the equinox is subject to both precession and nutation, and the decision to include the effects of one or both of these results in slightly different frames. Precession is the cyclic movement of the first point of Aries about the celestial sphere, which has a period of roughly 26,000 years. Nutation adds subtler oscillations on top of the mean movement of that precession.

**GEI2000**

GEI2000 creates a fully inertial frame by defining the first point of Aries and the Earth’s spin axis for a specific epoch, the J2000 reference epoch (2000-01-01T12:00:00 TT (Terrestrial Time); 2000-01-01T11:58:55.816 UTC). The SPICE system has a built-in reference frame, called J2000, which does just this, and this is what TRACERS uses for GEI2000.

**GEI_MOD**

The term ‘Mean-of-date’ generally refers to a frame where only precession is used. GEI_MOD is the GEI frame in that case. SPICE implements this by adding the effects of a precession model to J2000.

**GEI_TOD**

The term ‘True-of-date’ adds on the nutation correction, and is the actual best representation for the first point of Aries at any given epoch. GEI_TOD is the GEI frame in that case. SPICE implements this by adding the effects of both precession and nutation models to J2000.

#### A.1.2 Geographic (GEO) Frame

The Geographic frame is an Earth-fixed frame that uses the rotation axis of the Earth and a 0 degrees longitude reference meridian as the defining directions.

**Table:**

Z_GEO_	Along the spin axis of the Earth, pointing northward.
X_GEO_	Direction from the Earth’s center, in the plane perpendicular to the spin axis (the equatorial plane), that goes through the 0 degrees longitude reference meridian (commonly referred to as the Greenwich meridian).
Y_GEO_	Z_GEO_ × X_GEO_.

The SPICE system provides a high-precision ITRF93 Earth frame in the form of a regularly updated binary ephemeris file. TRACERS uses this ITRF93 frame as GEO.

#### A.1.3 Geocentric Solar Ecliptic (GSE) Frame

The Geocentric Solar Ecliptic frame uses the Earth-Sun line as the primary defining vector, and the relative velocity vector between the Earth and the Sun as the secondary vector.

**Table:**

X_GSE_	Direction of the geometric vector from the Earth to the Sun.
Z_GSE_	Direction of X_GSE_ × V_SE_ (V_SE_ = geometric velocity vector of the Sun as seen from the Earth).
Y_GSE_	Z_GSE_ × X_GSE_.

Equivalences:

X_GSE_ = X_GSM_

#### A.1.4 Geomagnetic (MAG) Frame

The Geomagnetic frame uses the Earth’s Centered Dipole (CD) axis, as defined by the IGRF-14 model, as the primary defining vector and the Earth’s rotation axis as the secondary vector. The frame is specified by a SPICE CK (attitude) kernel that encodes the orientation of a “virtual instrument” at the center of the Earth, and is defined by:

**Table:**

Z_MAG_	Along the vector from the center of the Earth through the north pole of the IGRF-14 CD.
Y_MAG_	In the direction of Z_GEO_ × Z_MAG_.
X_MAG_	Y_MAG_ × Z_MAG_.

This CK kernel is created by using the first three IGRF-14 coefficients to calculate the GEO locations of the CD north poles (Koochak and Fraser-Smith 2017) at a discrete set of epochs, with a cadence of 1 day. These are then used to calculate quaternions that transform from the GEO to the MAG frame at each epoch. This MAG frame is obtained by first rotating the GEO frame about the Z_GEO_ axis by the GEO longitude of the north CD pole, and then rotating about the resulting Y-axis by the GEO latitude of the north CD pole. Finally, these quaternions are processed by the msopck utility program provided with SPICE, generating a CK kernel.

The current MAG CK kernel was created using the IGRF-14 model. That model is considered definitive through 2019, provisional for 2020 through 2024, and is a predictive extrapolation for 2025 through 2029. Therefore, this MAG CK kernel has the same validity ranges.

Equivalences:

Z_MAG_ = Z_SM_

#### A.1.5 Geocentric Solar Magnetic (GSM) Frame

The Geocentric Solar Magnetic frame uses the Earth-Sun line as the primary defining vector, and the Earth’s Centered Dipole axis as the secondary vector.

**Table:**

X_GSM_	Direction of the geometric vector from the Earth to the Sun.
Y_GSM_	In the direction of Z_MAG_ × X_GSM_
Z_GSM_	X_GSM_ × Y_GSM_.

Equivalences:

X_GSM_ = X_GSE_

Y_GSM_ = Y_SM_

#### A.1.6 Solar Magnetic (SM) Frame

The Solar Magnetic frame uses the Earth’s Centered Dipole axis as the primary vector and the Earth-Sun line as the secondary vector.

**Table:**

Z_SM_	Along the vector from the center of the Earth through the north pole of the IGRF-14 CD.
Y_SM_	In the direction of Z_SM_ × R_ES_ (R_ES_ = geometric Earth-to-Sun vector).
X_SM_	Y_SM_ × Z_SM_.

Equivalences:

Y_SM_ = Y_GSM_

Z_SM_ = Z_MAG_

### A.2 Spacecraft-Specific Frames

#### A.2.1 TRACERS Spacecraft Coordinate System (TSCS) Frame

The TRACERS Spacecraft Coordinate System frame is the fundamental body-fixed frame for each spacecraft. The origin of this frame is at the geometric center of the launch vehicle adaptor ring on the separation interface plane. The frame is defined by the theoretical spacecraft spin axis and by the directions through the theoretical mount points for the Electric Field Investigation (EFI) antennas 1 and 3. Theoretical in all these cases meaning the zero-build-error design values.

**Table:**

Z_TSCS_	Along the theoretical design spin axis of the spacecraft.
Y_TSCS_	In the direction of Z_TSCS_ × A_EFI1_ (A_EFI1_ = vector from the TSCS origin through the theoretical design EFI1 antenna) (nominally along the theoretical EFI3 antenna).
X_TSCS_	Y_TSCS_ × Z_TSCS_ direction (nominally along the theoretical EFI1 antenna.

#### A.2.2 TRACERS Spinning Body (TSB) Frame

The TRACERS Spinning Body frame is a body-fixed spacecraft frame that uses the actual measured spin axis of the spacecraft as its primary defining vector, instead of the geometric Z_TSCS_ axis. In the ideal case that the principal moment of inertia of the spacecraft aligns with the Z_TSCS_ axis, the TSB frame is identical to the TSCS frame. This frame is specified by a SPICE CK kernel that is created using the angular velocity vectors provided by the spacecraft Guidance, Navigation and Control (GNC) system at a nominal 1-second cadence. The frame axes are defined by:

**Table:**

Z_TSB_	Along the direction of the positive angular velocity vector of the spacecraft.
Y_TSB_	Z_TSB_ × X_TSCS_ direction.
X_TSB_	Y_TSB_ × Z_TSB_ direction.

#### A.2.3 North East Center (NEC) Frame

This is an implementation of a spacecraft nadir-pointing frame, copying the North East Center (NEC) frame that was used on Swarm. A SPICE implementation of that frame uses the spacecraft-to-Earth vector as the primary vector and the GEO Z axis as the secondary vector.

**Table:**

Z_NEC_	Direction of vector from spacecraft to the center of the Earth.
Y_NEC_	In the direction Z_NEC_ × Z_GEO_ (eastward).
X_NEC_	Y_NEC_ × Z_NEC_.

#### A.2.4 Field-Aligned Coordinates (FAC) Frame

The Field-Aligned Coordinates frame uses the IGRF-14 B-field direction at the spacecraft location as the primary defining vector. The frame is specified by a SPICE CK (attitude) kernel that encodes the orientation of a “virtual instrument” on the TRACERS spacecraft, and is defined by:

**Table:**

Z_FAC_	Along the IGRF-14 model magnetic field at the spacecraft location.
Y_FAC_	In the direction of Z_FAC_ × R_Es_ (R_Es_ = Earth-to-spacecraft vector).
X_FAC_	Y_FAC_ × Z_FAC_.

This CK kernel is created along with each of the other SPICE kernels as each block of definitive spacecraft ephemeris is received by the SOC. The code makes use of the GEOPACK library to calculate the IGRF B-field at the tabulated spacecraft positions, and then constructs rotations from GEO to FAC for each position using the above definition of FAC. This sequence of rotations is then encoded within the CK kernel using the SPICE msopck utility program.

CAUTION: This frame is valid ONLY for calculations done at the location of the spacecraft, as the IGRF vectors are calculated specifically at that location, at the epoch when the spacecraft is there, according to the available ephemerides.

Equivalences:

Z_FAC_ = Z_FVC_

Z_FAC_ = Z_TFS_

#### A.2.5 Field Velocity Coordinates (FVC) Frame

The Field Velocity Coordinates frame is a second frame that uses the IGRF B-field direction at the spacecraft as the primary defining vector. In this case though, the secondary defining vector is the spacecraft velocity, as measured in the GEO frame.

**Table:**

Z_FVC_	Along the IGRF-14 model magnetic field at the spacecraft location.
Y_FVC_	Direction of Z_FVC_ × V_sE_ (V_sE_ = spacecraft-velocity-relative-to-Earth vector).
X_FVC_	Y_FVC_ × Z_FVC_.

Equivalences:

Z_FVC_ = Z_FAC_

Z_FVC_ = Z_TFS_

#### A.2.6 TRACERS Field Sun (TFS) Frame

The TRACERS Field Sun frame is the third frame that uses the IGRF B direction as the primary defining vector. It uses the spacecraft-to-Sun direction as the secondary vector.

**Table:**

Z_TFS_	Along the IGRF-14 model magnetic field at the spacecraft location.
Y_TFS_	Direction of Z_TFS_ × R_sS_ (R_sS_ = spacecraft-to-Sun vector).
X_TFS_	Y_TFS_ × Z_TFS_ (roughly sunward).

When the spacecraft is in the Region of Interest, and the Z_TSCS_ axis is aligned with the LABF vector, the TFS and TSS (Sect. A.2.7) frames should be very similar. This will not be true in general, however.

Equivalences:

Z_TFS_ = Z_FAC_

Z_TFS_ = Z_FVC_

#### A.2.7 TRACERS Spin Sun (TSS) Frame

The TRACERS Spin Sun frame uses the spacecraft spin axis as the primary defining vector, and the spacecraft-to-Sun direction as the second vector.

**Table:**

Z_TSS_	Along the spacecraft spin axis, as given by Z_TSB_.
Y_TSS_	Direction of Z_TSS_ × R_sS_ (R_sS_ = spacecraft-to-Sun vector).
X_TSS_	Y_TSS_ × Z_TSS_ (roughly sunward).

Note that nominally, the Z_TSS_ axis should be very close to the Z axis in the spacecraft body frame (Z_TSCS_).

When the spacecraft is in the Region of Interest, and the Z_TSCS_ axis is aligned with the LABF vector, the TFS and TSS frames should be very similar. This will not be true in general, however.
